# Laparoscopy is an available alternative to open surgery in the treatment of perforated peptic ulcers: a retrospective multicenter study

**DOI:** 10.1186/s12893-018-0413-4

**Published:** 2018-09-25

**Authors:** Antonino Mirabella, Tiziana Fiorentini, Roberta Tutino, Nicolò Falco, Tommaso Fontana, Paolino De Marco, Eliana Gulotta, Leonardo Gulotta, Leo Licari, Giuseppe Salamone, Irene Melfa, Gregorio Scerrino, Massimo Lupo, Armando Speciale, Gianfranco Cocorullo

**Affiliations:** 1 O.U. of Emergency and General Surgery of “Villa Sofia” Hospital, Palermo, Italy; 2 O.U. of Emergency and General Surgery of “Cervello” Hospital, Palermo, Italy; 3 Department of Surgical, Oncological and Stomatological Disciplines, University of Palermo, Palermo, Italy

**Keywords:** Peptic ulcer perforation, Laparoscopy, Stomach, Aged

## Abstract

**Background:**

Perforated peptic ulcers (PPU) remain one of the most frequent causes of death. Their incidence are largely unchanged accounting for 2–4% of peptic ulcers and remain the second most frequent abdominal cause of perforation and of indication for gastric emergency surgery. The minimally invasive approach has been proposed to treat PPU however some concerns on the offered advantages remain.

**Methods:**

Data on 184 consecutive patients undergoing surgery for PPU were collected. Likewise, perioperative data including shock at admission and interval between admission and surgery to evaluate the Boey’s score.

It was recorded the laparoscopic or open treatments, the type of surgical procedure, the length of the operation, the intensive care needed, and the length of hospital stay.

Post-operative morbidity and mortality relation with patient’s age, surgical technique and Boey’s score were evaluated.

**Results:**

The relationship between laparoscopic or open treatment and the Boey’s score was statistically significant (*p* = 0.000) being the open technique used for the low-mid group in 41.1% and high score group in 100% and laparoscopy in 58.6% and 0%, respectively. Postoperative complications occurred in 9.7% of patients which were related to the patients’ Boey’s score, 4.7% in the low-mid score group and 21.4% in the high risk score group (*p* = 0.000). In contrast morbidity was not related to the chosen technique being 12.8% in open technique and 5.3% in laparoscopic one (*p* = 0.092, *p* > 0.05).

30-day post-operative mortality was 3.8% and occurred in the 0.8% of low-mid Boey’s score group and in the 10.7% of the high Boey’s score group (*p* = 0.001). In respect to the surgical technique it occurred in 6.4% of open procedures and in any case in the Lap one (*p* = 0.043). Finally, there was a statistically significant difference in morbidity and mortality between patients < 70 and > 70 years old (*p* = 0.000; *p* = 0.002).

**Conclusions:**

Laparoscopy tends to be an alternative method to open surgery in the treatment of perforated peptic ulcer. Morbidity and mortality were essentially related to Boey’s score. In our series laparoscopy was not used in high risk Boey’s score patients and it will be interesting to evaluate its usefulness in high risk patients in large randomized controlled trials.

## Background

Perforated gastric ulcers remain one of the most frequent causes of death and disability worldwide.

The use of more tolerable drugs to control hyperacidity (H2 antagonists, proton pump inhibitors) and the treatments for eradication of helicobacter pylori infection (HP) enabled to reduce the rates of acid-reductive surgery (vagotomy, gastric resection) and re-intervention surgery due to recurrences.

The incidence of bleeding ulcers and its related mortality have decreased and its management – mainly guided by endoscopy and interventional radiology - have largely substituted surgery [1].

In contrast the incidence of perforated peptic ulcers (PPU) are largely unchanged, counting 2–4% of peptic ulcers which remain the second most frequent cause of abdominal perforation that requires surgery as well as the most frequent indication for gastric emergency surgery [1–3].

Seventy per cent of deaths from peptic ulcer disease are the result of perforation [3] and these should probably be attributed to the increasing use of anti-inflammatory drugs (NSAIDs) especially among aged patients with considerable co-morbidities for whom unchanged high morbidity rate (up to 50%) and mortality rate (up to 25%) still occur [4].

The minimal invasive approach has been proposed to treat PPU as well as other surgical emergencies, increasing the ability to tolerate the treatment among patients. In most centers the rate of laparoscopic management has gradually increased along with the improvement of technical skills [1–15].

## Methods

This study has assessed 184 consecutive patients undergoing surgery for PPU from 2006 to 2016 in three of the four major emergency surgery centers of Palermo (700.000 inhabitants), the Emergency and General Surgery O.U. of the University Hospital, the Emergency and General Surgery O.U. of “Villa Sofia” Hospital and the Emergency and General Surgery O.U. of “Cervello” Hospital.

The patients were identified by the diagnostic code on admission of ICD-9: 531.1, 531.5, 532.1, 532.5, 533.1, 533.5, recording demographical data including age, sex and ASA.

The analysis included the perioperative data including “shock at admission” and “time between admission and surgery” to evaluate the Boey’s score.

Also recorded the surgical procedures used, laparoscopic (Lap) or open, the type of surgical procedure, the length of the operation, the intensive care unit (ICU) needed and the lengths of hospital stay.

Post-operative morbidity and mortality were examined, while their relation with patient’s age, surgical technique and Boey’s score evaluated.

### Statistical analysis

Descriptive data are presented as percentage, mean ± standard deviation [SD] for parametric data and median, range and/or 95% confidence interval (CI) for non-parametric data.

The relationship between post-operative complications and mortality with age, Boey’s score and surgical technique were analyzed using the Pearson’s chi-square test or Fisher’s exact test. A *p*-value of < 0,05 was considered to be statistically significant.

Statistical analysis was conducted using SPSS software (SPSS, Chicago, Illinois, USA).

## Results

One hundred and eighty-four patients (61 female [range 22–91; mean 62 years], 123 male [range 21–88; mean age 59 years], M/F 3.1/1) underwent abdominal surgery for PPU between 2006 and 2016.

The estimated incidence in our district was 0.003%.

Median ASA score was 3. Shock at admission was reported in 64 patients (34.7%) while the time between the manifestation of symptoms and surgery was > to 24 h in 56 patients (30.4%).

One hundred twenty-eight patients showed a Boey’s score of 0–2 (low-mid), while 56 of 3(high) (Table 1).

**Table 1:** Patients’ Boey’s score

Boey’s score	Open	Lap
0–2	53	75
3	56	0

Open surgery was performed in 109 patients (59%) while 75 patients (41%) underwent laparoscopic surgery.

Contraindications to laparoscopy were Boey’s score > 2, multiple laparotomies, inadequate surgical skills.

No age restriction to Lap was adopted being Lap performed in patients that range between 35 and 79 years old.

Patients underwent ulcorraphy, ulcorraphy and omental patch or omental patch according to the diameter of the perforation (> or < 1 cm) and to the friability of the tissue surrounding the ulcer (Table 2).

**Table 2:** Patients’ surgical management

Surgical treatment	Open	Lap
Ulcorraphy	86	64
ulcorraphy and omental patch	20	9
omental patch only	3	2
Tot.	109	75

Median length of the surgical procedure was 70 (50–125) minutes for Lap technique and 52 (38–80) minutes for the open one.

Conversion rate was 26% (20 patients) and was due to adhesions or diffuse peritonitis.

ICU admission was required for 30 pts. (16%).

The mean length of hospital stay was 9 ± 4 days.

The relationship between Lap and Open to the different Boey’s scores was analyzed and there was a statistically difference between the two independent binomial proportions being open technique used for the low-mid group and high score group in 41.4% and 100%, respectively, while lap technique was used in 58.6% and 0% (*p* = 0.000).

Postoperative complications occurred in 18 patients (9.7%) (Table 3).

**Table 3:** Post-operative complications and related outcomes

	Type of surgery	Post-operative complications (10%)	Complication Treatment	Outcome
1	Lap	Acute pulmonary edema	ICU admission	Good
2	Lap	Fistula (low output)	Conservative	Good
3	Lap	Fistula (low output)	Conservative	Good
4	Lap	Fistula (high output)	Surgery	Good
5	Open	bronchopulmonary infection	ICU admission	Good
6	Open	bronchopulmonary infection	Conservative	Good
7	Open	IMA	ICU admission	Exitus
8	Open	gastric hemorrhage	Endoscopic	Good
9	Open	gastric hemorrhage	ICU admission	Exitus
10	Open	Septic Shock, MOF	ICU admission	Exitus
11	Open	bronchopulmonary infection	Conservative	Good
12	Open	bronchopulmonary infection	Conservative	Good
13	Open	IMA	ICU admission	Good
14	Open	bronchopulmonary infection	Conservative	Good
15	Open	IMA	ICU admission	Exitus
16	Open	Septic Shock	ICU admission	Exitus
17	Open	Septic Shock, MOF	ICU admission	Exitus
18	Open	Septic Shock, MOF	ICU admission	Exitus

The relationship between patients’ Boey’s score and morbidity revealed that 6 patients (4.7%) in the low-mid score group and 12 patients (21.4%) in the high risk score group had post-operative complications. A statistically significant difference in proportions of .167, *p* = 0.000 was found.

Morbidity was 12.8% (14/109) in open technique while 5.3% (4/75) in lap approach and the difference between the two independent binomial proportions was not statistically significant (*p* = 0.092, *p* > 0.05).

30-day post-operative mortality was 3.8% and causes arise from gastrointestinal bleeding to myocardial infarction (IMA) and septic shock with multiorgan failure (MOF) (Table 4).

**Table 4:** Post-operative mortality

Cases	Type of surgery	Post-operative days	Age (years)	Cause
1	Open	I	87	septic shock, MOF
2	Open	II	68	septic shock, MOF
3	Open	V	60	septic shock, MOF
4	Open	VIII	75	Gastric hemorrhage
5	Open	VIII	67	IMA
6	Open	X	83	IMA
7	Open	XV	79	septic shock, MOF

In respect to mortality low-mid Boey’s score group showed 0.8% (1/128) while high Boey’s score group showed 10.7% (6/56) and there was a statistically significant difference in proportions of .099, *p* = 0.001.

Mortality was 6.4% (7/109) in open technique conversely no deaths occurred in the Lap one. Due to small sample size, Fisher’s exact test was run. There was a statistically significant difference between the two independent binomial proportions (*p* = 0.043).

Patients older than 70 years were 9% (18 pts.), among them complications arose in 39% (7 pts.) and 30-day mortality was 22% (4pts.). There was a statistically significant difference in morbidity and mortality between patients < 70 and > 70 years old (*p* = 0.000; *p* = 0.002).

## Discussion

PPU diagnosis is based on clinical history, clinical findings and instrumental exams.

The reported estimated incidence is 1.5–3%, with a lifetime prevalence of 5% and a mortality rate ranging from 1.3 to 20% [16]. We considered patients operated in three of the four major hospitals of the district and our estimated incidence has been lower than reported data being 0.003%.

CT scan of the abdomen tends to be the most reliable exam in hemodynamically stable patients allowing also the identification of the perforation site [17, 18].

Several predictive scores were proposed over time for PPU even if most of them, as the Mannheim Peritonitis Index, are general scores often needing operative data.

The Boey’s score, a specific PPU score, due to its simplicity and high predictive value was largely used, with the following three parameters: state of shock on admission (systolic blood pressure < 90 mmHg), ASA III–V (presence of severe comorbidities), and duration of symptoms (> 24 h) [19, 20].

While many papers reported a relation between the Boey’s score and mortality only [1, 18, 21–23], Lohsiriwat reported a progressive increase in both morbidity and mortality related to increasing Boey’s score (11%, 47%, 75% and 77% for morbidity and of 1%,8%,33% and 38% for mortality) [19].

We analyzed the relationship between morbidity and mortality and low-mid (0–2) or high (3) Boey’s score showing a statistically significant relation between those (*p* = 0.000; *p* = 0.001). Morbidity was in our series 4.7% for Boey’s score 0–2 and 21.4% for Boey’s score 3 while mortality was 0.8% and 10.7%, respectively. Our morbidity rates were inferior to those reported by Lohsiriwat, this could be due to exclusion of wound infections from the outcomes that in this type of contamined or dirty operation can reach 15–40%.

Laparoscopy in the management of PPU has increased also due to the large diffusion of adequate skills among surgeons, as it happened to other surgical procedures that are actually mainly managed by minimal invasive approach [24, 25].

A recent Italian survey on the laparoscopic technique in the acute abdomen, published in 2016, shows that about 70% of the participant centers have modified the management of duodenal perforation in recent years comparing to the data from a 2012 Italian consensus (SICE-ACOI-SIC-SICUT-SICOP-EAES). Actually, more than 50% of gastro-duodenal perforations are treated by laparoscopy [25, 26].

A Cochrane review on the use of laparoscopic repair for PPU including three RCTs have found no statistically significant differences between laparoscopic and open surgery in the proportion of abdominal septic complications, pulmonary complications and number of septic abdominal complications. Authors stated that laparoscopic surgery results are not clinically different from those of open surgery [2, 4, 27, 28].

Contraindication to the laparoscopic approach were shock at admission, high Boey’s score, concomitant other ulcers complications, large perforations, technical difficulties, previous upper abdominal operations and serious associated cardiopulmonary diseases [28].

We valued as contraindication to laparoscopy: Boey’s score > 2, multiple laparotomies and inadequate surgical skills.

According to Berteleff, the choice of the surgical technique depends on lesion characteristics: if margins are edematous, friable, and/or difficult to mobilize, the repair can be limited to an omental patch, eventually associated with one or more sealant devices, while margins tend to be easily brought together without tension, direct suturing can be sufficient with or without omentoplasty [4].

Siu found 21.5% of conversion and causes of conversion: ulcers > 1 cm, technical difficulties and unidentifiable perforations [29].

In our series, main causes of conversion were adhesions and diffuse peritonitis while, on the basis of the diameter of the ulcer, the surgical repair was tailored looking at the dimension not necessarily as a reason to convert as suggested by Berteleff [4].

Lau demonstrated that laparoscopic repair of PPU confers short-term benefits in terms of postoperative pain and wound morbidity and that it is safe and effective as open repair [28].

In this regards our data showed that patients operated with open technique have higher morbidity rates (12.8% vs 5.3%) even if not statistically significant difference was proved (*p* = 0.092, *p* > 0.05). However, in our opinion this high rate of morbidity should be correlated to the inclusion in the open group of patients with higher Boey’s score who were not managed laparoscopically.

These data were confirmed by our patient’s mortality rate that were 6.4% in open surgery while 0% in laparoscopic surgery with a statistically significant difference (*p* = 0.043).

The progressive increase of elderly population suffering from PPU often with frank peritonitis may represent an obstacle to a more rapid discharge and to an immediate resumption of normal activities [29].

In our series no age restriction to Lap was adopted, it was actually performed in patients with ages ranged between 35 and 79 years old and our data showed a statistically significant difference in morbidity and mortality between patients < 70 and > 70 years old (*p* = 0.000; *p* = 0.002) irrespective of the used technique.

## Conclusions

In conclusion findings show that laparoscopy could be a possible alternative to open surgery when treating perforated peptic ulcer, and literature data don’t show a statistically significant difference from open surgery.

Morbidity and mortality resulted statistically related to Boey’s score, while only mortality was statistically related to the surgical technique being laparoscopy not used in high risk Boey’s score patients.

In this regard, it would be interesting to evaluate the safety and usefulness of laparoscopic surgery in high risk patients.

However, there would be the need of large RCTs to verify those outcomes.

## Abbreviations

ASA: American society of anestesiologists
HP: helicobacter pylori infection
ICD: international classification of diseases
ICU: intensive care unit
IMA: myocardial infarction
Lap: laparoscopy
MOF: multiorgan failure
NSAIDs: anti-inflammatory drugs
PPU: perforated peptic ulcers
